# In Silico Integration Approach Reveals Key MicroRNAs and Their Target Genes in Follicular Thyroid Carcinoma

**DOI:** 10.1155/2019/2725192

**Published:** 2019-03-28

**Authors:** Shengqing Hu, Yunfei Liao, Juan Zheng, Luoning Gou, Anita Regmi, Mohammad Ishraq Zafar, Lulu Chen

**Affiliations:** Department of Endocrinology, Union Hospital, Tongji Medical College, Huazhong University of Science and Technology, Wuhan 430022, China

## Abstract

To better understand the molecular mechanism for the pathogenesis of follicular thyroid carcinoma (FTC), this study aimed at identifying key miRNAs and their target genes associated with FTC, as well as analyzing their interactions. Based on the gene microarray data GSE82208 and microRNA dataset GSE62054, the differentially expressed genes (DEGs) and microRNAs (DEMs) were obtained using R and SAM software. The common DEMs from R and SAM were fed to three different bioinformatic tools, TargetScan, miRDB, and miRTarBase, respectively, to predict their biological targets. With DEGs intersected with target genes of DEMs, the gene ontology (GO) and Kyoto Encyclopedia of Genes and Genomes (KEGG) pathway enrichment analysis were performed through the DAVID database. Then a protein-protein interaction (PPI) network was constructed by STRING. Finally, the module analysis for PPI network was performed by MCODE and BiNGO. A total of nine DEMs were identified, and their function might work through regulating hub genes in the PPI network especially KIT and EGFR. KEGG analysis showed that intersection genes were enriched in the PI3K-Akt signaling pathway and microRNAs in cancer. In conclusion, the study of miRNA-mRNA network would offer molecular support for differential diagnosis between malignant FTC and benign FTA, providing new insights into the potential targets for follicular thyroid carcinoma diagnosis and treatment.

## 1. Introduction

Follicular thyroid carcinoma (FTC) is the second most common thyroid malignancy after papillary thyroid carcinoma [1]. The advances in diagnostic techniques have made it easier to identify thyroid malignancy in recent years [2]. Unfortunately, it is still difficult to distinguish the follicular architecture of a malignancy follicular thyroid carcinoma (FTC) from that of follicular thyroid adenoma (FTA), which enjoys a better prognosis [3]. Currently, fine-needle aspiration (FNA) is the most reliable, widely used, and cost-effective preoperative test for initial evaluation of thyroid nodules, but it has a low accuracy for both FTC and FTA [4]. Understanding the pathogenesis of FTC at the molecular level significantly contributes to its diagnosis and therapy, particularly for an accurate differentiation between FTC and FTA.

There is emerging evidence that has demonstrated the participation of multiple genes and cellular pathways in the occurrence and development of FTC [5–7]. More importantly, as the endogenous noncoding small regulatory RNAs, microRNAs (miRNAs) have also indicated its role in numerous and wide-ranging biological processes, including cell proliferation, differentiation, development, apoptosis, pathogenesis disease resistance, tumorigenesis, and lipogenesis [8–10]. To date, however, a precise molecular mechanism for the FTC progression remains unclear. Though many studies using microarray technology have explored the differentially expressed microRNAs (DEMs) or differentially expressed genes (DEGs) between FTC and FTA [11–14], these results show little overlap since the samples are limited and the results contain significant false-negatives. Recent two works give more precise results for DEMs [15] and DEGs [16] by using multiple databases for cross-validation. However, they have not studied inverse interaction relationships between DEMs and their target DEGs, particularly the pathways in the interaction network. Since the regulatory control network existing between miRNAs and mRNAs plays an intricate role in biological pathways [17], an integrated analysis of the interaction relationship would benefit a lot for understanding their underlying molecular mechanisms.

In this study, we screened out DEMs and DEGs between FTC and FTA via microarray technology and integrated bioinformatics analyses. Intersection genes between DEGs and DEMs-target genes were extracted, classified, and extensively analyzed. We explored the enrichment in Gene Ontology (GO) and Kyoto Encyclopedia of Genes and Genomes (KEGG) pathway terms, constructed miRNA-target gene network, and investigated protein-protein interaction (PPI) network along with its key hub genes and significant module. We found several differentially expressed mRNAs and miRNAs and revealed their inverse relationships that might promote the progress of FTC. More importantly, we showed that mRNAs targeted by miRNAs are mainly enriched in the PI3K-Akt signaling pathway and microRNAs in cancer. Our results provide the potential candidate biomarkers for FTC diagnosis, pathogenesis, and drug targets, shedding new light on its development at the molecular level.

## 2. Materials and Methods

### 2.1. Microarray Data

Gene Expression Omnibus (GEO, http://www.ncbi.nlm.nih.gov/geo) is a public repository for curated gene expression datasets, original series, and platform records. From GEO we obtained miRNA expression profile GSE62054 [15] which contains eight adenoma samples and 17 FTC samples, and gene expression profile GSE82208 [16] which includes 24 thyroid adenoma samples and 27 FTC samples. To give a comparison, we also obtained RNA-Seq data for FTA and FTC from European Nucleotide Archive (ENA, PRJEB11591) and The Cancer Genome Atlas (TCGA, TCGA-BJ-A0ZF) to identify differentially expressed mRNAs and miRNAs.

### 2.2. Identification of DEMs and DEGs

We used software R (version 3.5.0, https://www.r-project.org/) and packages from Bioconductor (http://www.bioconductor.org/) to conduct significance analysis of DEGs and DEMs between FTC samples and FTA samples, respectively. The microarray data were first preprocessed using the algorithm “RMA,” which contains background adjustment and normalization with the quantile method. Then the Moderated T statistic approach was applied to select significant DEGs or DEMs with “limma” package [18] of Bioconductor. Finally, DEGs or DEMs were annotated through annotation table downloaded from the GEO website. P values were adjusted by the default Benjamini & Hochberg (BH) false discovery rate method. The adj.P value < 0.05 and |logFC| > 1 were considered as significantly different for DEGs, while adj.P value < 0.05 and |logFC| > 0.8 were used for DEMs. We also choose a set of different values of |logFC| to show their impacts on the final results. For the RNA-seq data from ENA, we downloaded three samples for each set of FTA and FTC. All the sequencing reads were aligned to the human reference genome (GRCh38) by software Tophat2. Then we used software HTSeq to calculate read counts for each gene, the results of which were used to find differentially expressed genes with “edgeR” package [19]. For the RNA-seq data from TCGA, we use another five samples from the above ENA database to find differentially expressed miRNA by using “edgeR” package as well. To ensure that the targets are not false positives, we used the Significance Analysis of Microarrays (SAM) (http://statweb.stanford.edu/~tibs/SAM/), a supervised learning software, to identify DEMs and DEGs. We set* Delta* value to 0.75 for miRNA and 1.25 for mRNA, respectively, such that we get a false discovery rate less than 0.01. The random number seed was 1234567, and the number of permutation was optimized to 100. The common results from R and SAM methods were selected as the final outcomes.

### 2.3. Integrated Analysis of DEMs and DEGs

TargetScan (http://www.targetscan.org/vert_71/), miRDB (http://www.mirdb.org/), and miRTarBase (http://mirtarbase.mbc.nctu.edu.tw/php/index.php) are three different integrated databases to predict the biological targets of miRNAs. We used all of them to retrieve the target genes of DEMs. The target genes shown in all three databases were selected. The selected target genes of DEMs which were found overlapping with the DEGs were designated as “intersection genes” and extracted via the online tool Venny 2.1 (http://bioinfogp.cnb.csic.es/tools/venny/index.html). The miRNA-intersection genes regulation network was constructed and visualized using Cytoscape [20]. The cumulative weighted context++ score < -0.1 was applied as the cut-off criteria. Targets with lowest context scores are the most representative ones. Therefore, we use context score to screen out target genes in order to obtain a more reliable result. A hierarchical clustering analysis of intersection genes was constructed using the online tool Morpheus (https://software.broadinstitute.org/morpheus/). Average linkage method was performed with one minus Pearson correlation. The heatmap was constructed based on the gene expression values in each sample.

### 2.4. Functional and Pathway Enrichment Analysis for Intersection Genes

Gene ontology (GO, http://www.geneontology.org/) is a framework for the model of biology, which describes gene functions and classifies them along three aspects: molecular function (MF), cellular component (CC), and biological process (BP) [21]. Kyoto Encyclopedia of Genes and Genomes (KEGG, http://www.genome.jp/kegg/) is a collection of the database for understanding high-level gene functions and utilities of the biological system [22]. The Database for Annotation, Visualization and Integrated Discovery (DAVID, https://david.ncifcrf.gov/home.jsp) is a tool that provides a comprehensive set of functional annotation tools to investigate the biological meaning behind a mass of genes [23]. To provide a function-level analysis for intersection genes, GO term enrichment analysis and KEGG pathway enrichment analysis were conducted using DAVID. We considered P value < 0.05 to have statistical significance and achieve significant enrichment.

### 2.5. PPI Network Construction and Modules Analysis for Intersection Genes

Search Tool for the Retrieval of Interacting Genes (STRING, https://string-db.org/) is a freely accessible biological database to evaluate protein-protein interaction (PPI) information. The STRING (version 10) contains 9,643,763 proteins from 2031 organisms. In order to figure out the interactive relationships among intersection genes, the DEGs were mapped to STRING with the confidence score > 0.7 set as the cut-off criterion. Then the PPI network was constructed and visualized using Cytoscape. Besides, the plug-in Molecular Complex Detection (MCODE) [24] was used to screen the most significant module of the PPI network with the following criteria: Max. Depth = 100, K-Core = 2, Mode Score Cutoff = 0.2, and Degree Cutoff = 2. Moreover, the function and pathway enrichment analysis were conducted for intersection genes in each module with the plug-in Biological Network Gene Ontology (BiNGO). A P value < 0.05 was considered to have significant differences. To give a comparison with the result from STRING, we also used the BioGRID, another interaction repository which stores a curated set of data, to illustrate PPI results.

## 3. Results

### 3.1. Identification of DEMs and DEGs in FTC

A total of 348 DEGs were identified from GSE82208 which contains 24 FTA samples and 27 FTC samples. Of these, 289 genes were upregulated, and 59 genes were downregulated. Meanwhile, a total of 9 DEMs were identified from GSE62054 using P value <0.05. Among them, 6 miRNAs were significantly downregulated (hsa-miR-7-2-3p, hsa-miR-7-5p, hsa-miR-130b-5p, hsa-miR-144-5p, hsa-miR-1179, and hsa-miR-328-3p), while the expression levels of the left miRNAs (hsa-miR-767-5p, hsa-miR-663b, hsa-miR-137) increased conversely in FTC (Table 1). The DEGs and DEMs identified from ENA and TCGA databases are shown in the Supplementary Tables S1 and S2, respectively. The results from different thresholds of |logFC| are shown in Supplementary Figure S1, and their GO and KEGG analyses are shown in the Supplementary Tables S3, S4, S5, and S6.

### 3.2. Integrated Analysis of DEMs and DEGs

**Figure 1:**
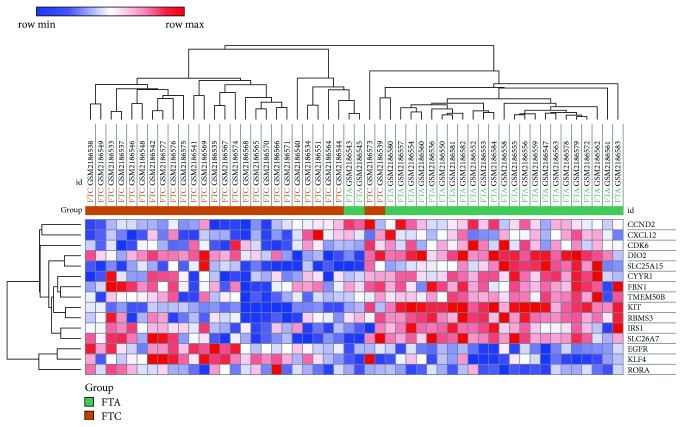
Hierarchical clustering analysis of intersection genes. Each row represents one of the intersection genes, and each column represents a tissue sampled from follicular thyroid tumors. FTA or FTC. Column “FTA” represents follicular thyroid adenoma, while column “FTC” represents tissue from follicular thyroid cancer. The heatmap was constructed based on gene expression values in each sample. Red and blue colors indicate higher expression and lower expression, respectively.

**Figure 2:**
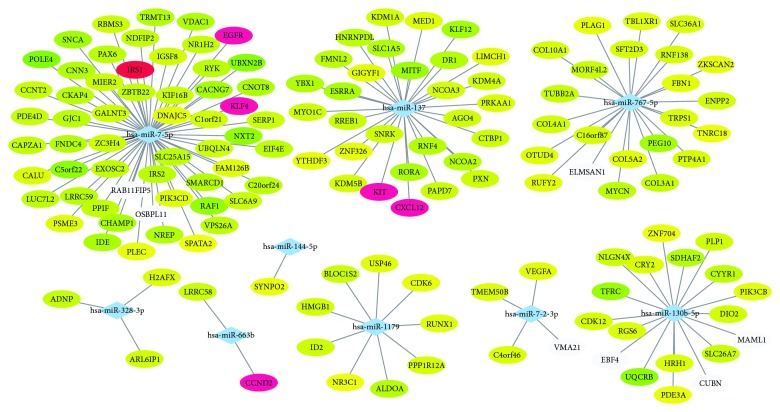
A network of DEMs and their common target genes. Diamonds represent aberrant miRNAs, and ellipses represent target genes. The red nodes are the hub genes screened in the subsequent PPI network, and the rest are ranked by cumulative weighted context++ score (a lower context++ score is greener).

The miRNAs could interact with their mRNA targets, resulting in the post-transcriptional suppression of their target genes. After predicting potential candidate targets of miRNAs using TargetScan, miRDB, and miRTarBase, a total of 456 target genes for 9 DEMs were identified. Table 1 shows the most probable target genes of DEMs. Furthermore, 15 intersection genes were found between target genes and DEGs. Among them, 12 genes were downregulated, and 3 genes were upregulated. A hierarchical clustering analysis of intersection genes revealed the differential expression in FTC compared to FTA (Figure 1). For further elucidation of interactions between the aberrant miRNAs considered herein and the intersection genes, the miRNA-intersection gene network was constructed using Cytoscape (Figure 2). The miRNAs are listed in Table 1.

### 3.3. Go Term Enrichment and KEGG Pathway Analyses of Interactions between the Potential Targets of Aberrant miRNAs and DEGs

To explore the biological functions of the 15 intersection genes concerning the regulation of FTC pathogenesis, we uploaded all intersection genes to DAVID to find out overrepresented GO categories and KEGG pathways. The information we obtained regarding biological function indicated that these 15 intersection genes were enriched in 5 Gene Ontology_Biological Process (GO_BP), 1 Gene Ontology_Cellular Component (GO_CC), and 4 Gene Ontology_Molecular Function (GO_MF) terms as well as 5 KEGG pathway terms (Table 2). Among these biological processes, the intersection genes were mainly associated with the regulation of phosphatidylinositol 3-kinase signaling. In the cell component (CC) ontology, the intersection genes were enriched in cyclin-dependent protein kinase holoenzyme complex. Besides, the molecular function (MF) analysis indicated that the intersection genes were significantly enriched in phosphatidylinositol-4,5-bisphosphate 3-kinase activity. Moreover, Table 3 shows the most significantly enriched pathways of the intersection genes through KEGG analysis. The genes were mainly enriched in the PI3K-Akt signaling pathway and microRNAs in cancer.

### 3.4. PPI Network Construction and Module Selection

**Figure 3:**
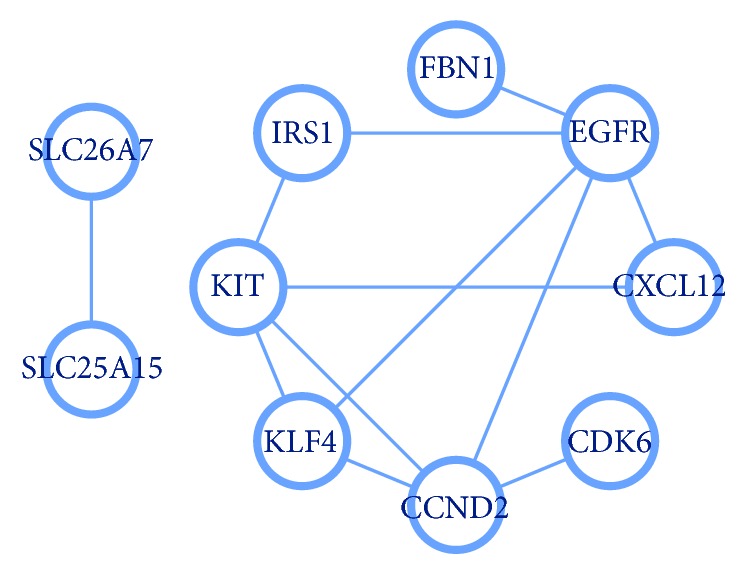
Protein-protein interaction networks for intersection genes.

**Figure 4:**
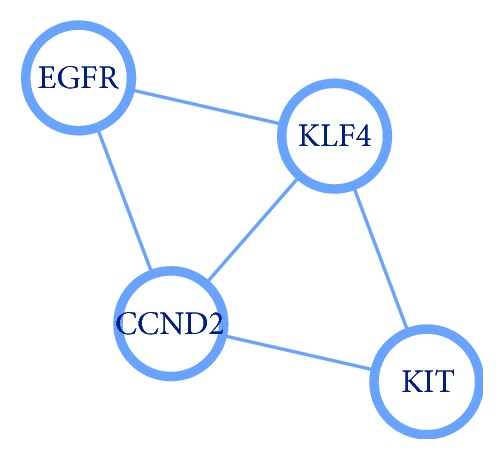
The most significant module of PPI network.

To understand the connection between intersection genes further, 15 intersection genes were used to construct the PPI network (Figure 3). Based on the information from the STRING database, the top 5 intersection genes (according to degrees) were screened out and selected as hub genes, which showed a strong association with other node proteins, as depicted in Table 4. For visualizing the connection between hub genes and their regulated miRNAs, hub genes were highlighted by red color in Figure 2. Among these hub genes, the EGFR showed the highest node degree 5, and its related miRNA was miR-7-5p. In addition, the most significant module was selected (Figure 4), and the corresponding GO enrichment analysis (Table 5) showed that the genes in this module are mainly associated with positive regulation of cyclin-dependent protein kinase activity. The PPI interaction derived from the BioGRID database is shown in Supplementary Figure S2. It showed that both EGFR and KIT appear in the top 2 hub genes.

## 4. Discussion

The distinction between a well-encapsulated, minimally invasive FTC and a benign follicular adenoma may be difficult. Since FTA tends to have a better prognosis [25], it is of great importance to understand the molecular mechanisms of the proliferation, apoptosis, and invasion of FTC. The miRNAs are a group of noncoding small RNAs that play an important role in regulating a spectrum of basic cellular processes [26]. Endogenous miRNAs interact with the 3′-UTR of target mRNA to suppress post-transcription of genes [27]. Our study results are consistent with previous reports [15, 16] that aimed at screening out DEGs or DEMs. However, these reports did not unleash or investigated the interaction mechanisms between the DEMs and their target DEGs, particularly the pathways in the interaction network. In this work, in order to improve the accuracy and reliability, we made use of multiple bioinformatics approaches and predicting databases to construct the miRNA-mRNA network, having reduced the false negative results at the most extent.

In this study, the gene expression profile data GSE82208 and microRNA expression profile GSE62054 were downloaded from the GEO database to identify DEGs and DEMs, respectively. It was identified that downregulated miRNAs include hsa-miR-7-2-3p, hsa-miR-7-5p, hsa-miR-1179, hsa-miR-130b-5p, hsa-miR-144-5p, and hsa-miR-328-3p, and upregulated miRNAs include hsa-miR-767-5p, hsa-miR-663b, and hsa-miR-137. There are 15 intersection genes between DEGs and DEMs target genes, of which two genes were upregulated while 13 were downregulated. The protein-protein interaction (PPI) network for intersection genes was screened out, with which top 5 hub genes and the most significant module were selected and analyzed for concerning biological functions. The top 5 hub genes in the PPI network were EGFR, KIT, CCND2, KLF4, and CXCL12.

Generally, there exists a negative correlation between miRNAs and their target genes. The miRNAs could downregulate the expression of the target genes by diminishing the stability transcription or inhibiting translation [27, 28]. The dysregulation of miRNAs plays a vital role in the pathogenesis of multiple cancer types, including FTC [29]. In this study, we identified 9 DEMs, including six downregulated and three upregulated miRNAs in FTC compared to FTA. The significantly downregulated miRNA miR-7-5p targets 2 hub genes EGFR and KLF4, whose expressions were upregulated accordingly. It is known that miR-7-5p functions as a tumor suppressor, inhibiting proliferation, migration, and invasion of thyroid papillary cancer cells. The epidermal growth factor receptor (EGFR) family of receptor tyrosine kinases lies at the head of complex signal transduction cascade that modulates cell proliferation, survival, adhesion, migration, and differentiation. We found that EGFR has the highest degree among all the miRNA-target genes (Table 4), which further validates its essential role in tumor progression and metastasis [30, 31]. We also suggest that downregulated miR-7-5p can significantly upregulate EGFR, which is consistent with previous studies showing that the knockdown of miR-7-5p could increase the expression of EGFR in dental pulp stem cells [32]. In addition, KLF4 was identified as a potential therapeutic target for eliminating ATC cells [33]. Our results support the hypothesis that miR-7-5p could regulate the development of FTC by targeting EGFR and KLF4.

Our study shows that KIT and CXCL12 are decreased in FTC, both of which might be regulated by the increased miR-137. We attribute our results to the fact that KIT signaling can promote cell proliferation and survival, while getting decreased in human follicular thyroid cancer [34, 35]. Moreover, CXCL12 might serve as an effective novel supplementary diagnostic marker for PTC [36]. As a comparison, it has been proved that miR-137 could downregulate KIT or CXCL12 in other cancers such as small cell lung cancer [37], acute myeloid leukemia [38], and glioblastoma [39]. However, recent research has shown that miR-137 was downregulated in thyroid cancer and inhibits proliferation and invasion by targeting EGFR [40], and it could act as a tumor suppressor in papillary thyroid carcinoma [41]. We believe that accumulating data would lead to a better understanding of the molecular regulation mechanism between miR-137 and KIT in FTC.

CCND2 is a promising target in differentiating FTC from FTA [42]. We suggest that it is downregulated and could be targeted by upregulated miR-663b. However, few works have studied about miR-663b, except for the fact that its downregulation could repress proliferation and induce apoptosis in osteosarcoma [43] or exert its tumor-suppressive function in the pancreatic cancer [44]. Our new results on the interactions between miR-663b and CCND2 would provide a new perspective to differentiate FTC from FTA.

Since each mRNA may be targeted by several miRNAs and participate in different biological functions, miRNA can affect the multiple biological processes and pathways through a miRNA-mRNA network. Our KEGG analysis showed that most of the intersection genes were enriched in the PI3K-Akt signaling pathway and microRNAs in cancer. Moreover, the analysis of the most significant module showed that the genes in this module were mainly associated with positive regulation of cyclin-dependent protein kinase activity. Among the hub genes which play a critical role in the biological process, both miR-7-5p and miR-137 could regulate 2 hub genes, and miR-663b could regulate one hub gene. It indicates the fact that one miRNA can target multiple genes, which has also been observed before [45]. Our results suggest that several pathways were regulated by a miRNA-mRNA network during the tumorigenesis and progression of FTC.

This study identified several key genes and miRNAs, as well as potential biomarkers in predicting the progression of FTC. We found that EGFR, KIT, CCND2, KLF4, and CXCL12 might be the key aberrant genes that figure prominently in the pathogenesis of FTC. The main dysregulated miRNA, including miR-7-5p, miR-663b, and miR-137, could regulate the expression of target hub genes and further affect the biological process of FTC. Note that we have applied the same analysis approaches on RNA-seq data to validate our results (i.e., ENA for DEG and TCGA for DEM). We found that most of our intersection genes also appear in the new experiments (Supplementary Table S1), while only a few common DEMs obtained (Supplementary Table S1). We think that the different databases indeed have an impact on finding differentially expressed miRNA and more experiments are needed. Overall, our results offered molecular support for the differential diagnosis between malignant follicular thyroid cancer and benign follicular thyroid adenoma. More importantly, it provided new insights into the potential targets for follicular thyroid carcinoma diagnosis and treatment.

## Figures and Tables

**Table 1:** Aberrant miRNAs and their most probable target genes.

miRNA_ID	Expression	P value	adj.P value	logFC	Most probable target genes
hsa-miR-7-2-3p	down-regulated	0.0001655	0.001986	-1.363672	VMA21, VEGFA, TMEM50B, QSER1, LMAN1

hsa-miR-767-5p	up-regulated	0.000997	0.0028464	0.820405	RNF138, SH3GLB1, PEG10, SFT2D3, MYCN

hsa-miR-7-5p	down-regulated	0.0011923	0.0036794	-1.922032	SNCA, EGFR, IRS1, KLF4, IRS2, RAF1

hsa-miR-663b	up-regulated	0.0016628	0.0038779	0.891045	CCND2, LRRC58

hsa-miR-130b-5p	down-regulated	0.0017059	0.0042245	-0.906782	NOTCH2, CGNL1, ANKRD28, TFRC, PLP1

hsa-miR-144-5p	down-regulated	0.0021107	0.0052289	-0.904925	SYNPO2

hsa-miR-1179	down-regulated	0.0021463	0.007189	-1.640942	CDK6, BLOC1S2, USP46, ID2, PPP1R12A

hsa-miR-137	up-regulated	0.0028163	0.0192025	1.039935	NCOA2, KIT, KDM1A, CTBP1, CXCL12, MITF, ESRRA,

hsa-miR-328-3p	down-regulated	0.0039217	0.0477905	-0.871384	H2AFX, ADNP, ARL6IP1

**Table 2:** Gene ontology analysis of intersection genes.

Category	Term	Count	P value	Benjamini	Genes
GOTERM_BP_DIRECT	GO: 0014066~regulation of phosphatidylinositol 3-kinase signaling	3	0.001364	0.128529	EGFR, KIT, IRS1

GOTERM_BP_DIRECT	GO: 0046854~phosphatidylinositol phosphorylation	3	0.001973	0.085255	EGFR, KIT, IRS1

GOTERM_BP_DIRECT	GO: 0048015~phosphatidylinositol-mediated signaling	3	0.0025	0.066478	EGFR, KIT, IRS1

GOTERM_BP_DIRECT	GO: 0008284~positive regulation of cell proliferation	4	0.003875	0.041446	EGFR, CCND2, KIT, IRS1

GOTERM_BP_DIRECT	GO: 0010628~positive regulation of gene expression	3	0.014438	0.29193	CDK6, KIT, KLF4

GOTERM_CC_DIRECT	GO: 0000307~cyclin-dependent protein kinase holoenzyme complex	2	0.011466	0.285394	CCND2, CDK6

GOTERM_MF_DIRECT	GO: 0046934~phosphatidylinositol-4,5-bisphosphate 3-kinase activity	3	8.55E-04	0.028534	EGFR, KIT, IRS1

GOTERM_MF_DIRECT	GO: 0005088~Ras guanyl-nucleotide exchange factor activity	3	0.002904	0.048361	EGFR, KIT, IRS1

GOTERM_MF_DIRECT	GO: 0004716~receptor signaling protein tyrosine kinase activity	2	0.007088	0.117378	EGFR, KIT

GOTERM_MF_DIRECT	GO: 0004714~transmembrane receptor protein tyrosine kinase activity	2	0.026689	0.299626	EGFR, KIT

**Table 3:** KEGG pathway analysis of intersection genes.

Term	Count	%	P value	Benjamini	Genes
hsa04151: PI3K-Akt signaling pathway	5	33.33333	6.41E-04	0.012844	EGFR, CCND2, CDK6, KIT, IRS1

hsa05206: MicroRNAs in cancer	4	26.66667	0.004956	0.071044	EGFR, CCND2, CDK6, IRS1

hsa05200: Pathways in cancer	4	26.66667	0.012004	0.114885	EGFR, CDK6, KIT, CXCL12

hsa04068: FoxO signaling pathway	3	20	0.012396	0.117275	EGFR, CCND2, IRS1

**Table 4:** Top 5 hub genes in PPI network and their related DEMs (node degree: the number of edges incident to the node; related miRNAs: miRNAs that target the gene).

Name	Expression	Node Degree	Related DEM
EGFR	up-regulated	5	miR-7-5p (down)

KIT	down-regulated	4	miR-137 (up)

CCND2	down-regulated	4	miR-663b (up)

KLF4	up-regulated	3	miR-7-5p (down)

CXCL12	down-regulated	2	miR-137 (up)

**Table 5:** GO enrichment analysis for the most significant module in PPI network.

GO-ID	P value	Corr. P value	Description	Genes in test set
45737	3.87E-06	1.69E-03	positive regulation of cyclin-dependent protein kinase activity	CCND2|EGFR

42127	1.23E-05	1.91E-03	regulation of cell proliferation	CCND2|KIT|KLF4|EGFR

45860	1.75E-05	1.91E-03	positive regulation of protein kinase activity	CCND2|KIT|EGFR

33674	1.96E-05	1.91E-03	positive regulation of kinase activity	CCND2|KIT|EGFR

51347	2.18E-05	1.91E-03	positive regulation of transferase activity	CCND2|KIT|EGFR

50679	5.53E-05	4.03E-03	positive regulation of epithelial cell proliferation	CCND2|EGFR

45859	6.68E-05	4.07E-03	regulation of protein kinase activity	CCND2|KIT|EGFR

43549	7.47E-05	4.07E-03	regulation of kinase activity	CCND2|KIT|EGFR

51338	8.37E-05	4.07E-03	regulation of transferase activity	CCND2|KIT|EGFR

79	1.03E-04	4.07E-03	regulation of cyclin-dependent protein kinase activity	CCND2|EGFR

## Data Availability

The microarray data used to support the findings of this study are available in the GEO, TCGA, and ENA repository.

## Supplementary Materials

Supplementary Materials Figure S1: identification results of differentially expressed genes or miRNAs under different thresholds of |logFC|. Figure S2: PPI network for intersection genes using the BioGRID database. Table S1: common genes between DEG_ENA and our intersection genes. Table S2: common miRNAs between DEM_TCGA and our DEM. Table S3: GO analysis for intersection genes selected with |logFC| > 0.8 for DEM, and |logFC| > 1.5 for DEG. Table S4: KEGG analysis for intersection genes selected with |logFC| > 0.8 for DEM, and |logFC| > 1.5 for DEG. Table S5: GO analysis for intersection genes selected with |logFC| > 1 for DEM, and |logFC| > 1 for DEG. Table S6: KEGG analysis for intersection genes selected with |logFC| > 1 for DEM, and |logFC| > 1 for DEG.
