# Supplementary material for: In Silico Integration Approach Reveals Key MicroRNAs and Their Target Genes in Follicular Thyroid Carcinoma
Source: Biomed Res Int. 2019 Mar 28;2019:2725192. doi: 10.1155/2019/2725192 (PMC6458921; doi:10.1155/2019/2725192)
Supplement: Supplementary Materials — Figure S1: identification results of differentially expressed genes or miRNAs under different thresholds of |logFC|. Figure S2: PPI network for intersection genes using the BioGRID database. Table S1: common genes between DEG_ENA and our intersection genes. Table S2: common miRNAs between DEM_TCGA and our DEM. Table S3: GO analysis for intersection genes selected with |logFC| > 0.8 for DEM, and |logFC| > 1.5 for DEG. Table S4: KEGG analysis for intersection genes selected with |logFC| > 0.8 for DEM, and |logFC| > 1.5 for DEG. Table S5: GO analysis for intersection genes selected with |logFC| > 1 for DEM, and |logFC| > 1 for DEG. Table S6: KEGG analysis for intersection genes selected with |logFC| > 1 for DEM, and |logFC| > 1 for DEG. [file 2725192.f1.pdf]

Figure S1 Identification results of differentially expressed genes or miRNAs under different thresholds of |logFC|.

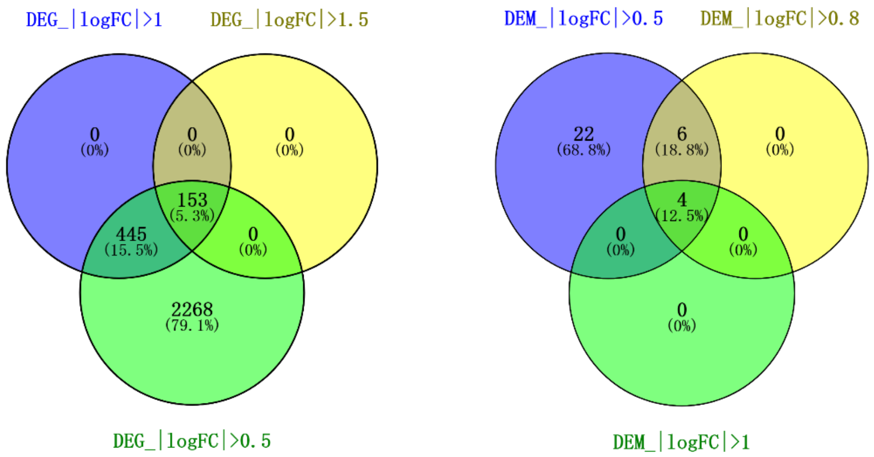

Figure S2 PPI network for intersection genes using the BioGRID database.

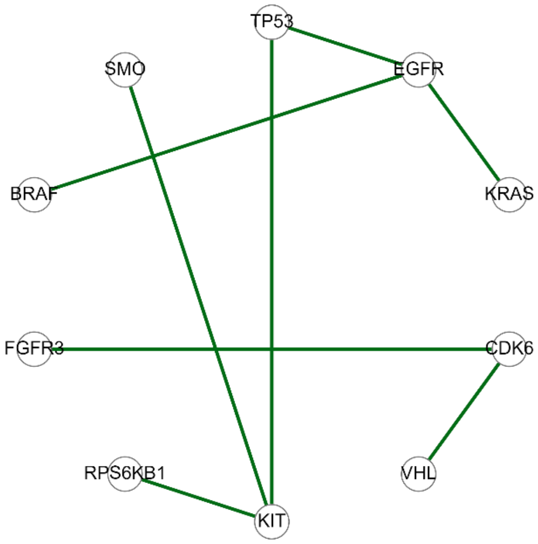

Table S1 Common genes between DEG_ENA and our intersection genes.

Gene.symbol	P.Value	adj.P value	logFC	Gene title
CCND2	2.71E-03	0.00657	-1.52	cyclin D2
CDK6	5.91E-03	0.008865	-1.12	cyclin dependent kinase 6
CXCL12	2.63E-02	0.0263	-1.4	C-X-C motif chemokine ligand 12
CYYR1	1.51E-04	0.001359	-1.21	cysteine and tyrosine rich 1
DIO2	3.55E-03	0.00657	-1.13	deiodinase, iodothyronine, type II
EGFR	1.78E-03	0.00657	1.16	epidermal growth factor receptor
FBN1	1.04E-02	0.0117	-1.07	fibrillin 1
KIT	3.65E-03	0.00657	-2.32	KIT proto-oncogene receptor tyrosine kinase
SLC26A7	7.68E-03	0.0098743	-1.29	solute carrier family 26 member 7

Table S2 Common miRNAs between DEM_TCGA and our DEM.

miRNA_ID	logFC	P value	adj.P value
hsa-miR-7-2-3p	-1.363672	0.000166	0.001986
hsa-miR-130b-5p	-0.906782	0.001706	0.0042245
hsa-miR-144-5p	-0.904925	0.002111	0.005229

Table S3 GO analysis for intersection genes selected with |logFC|>0.8 for DEM, and |logFC|>1.5 for DEG.

Category	Term	Count	P value	Genes
GOTERM_BP_DIRECT	GO: 0006520~cellular amino acid metabolic process	2	0.016559	SLC7A5, SLC25A15
GOTERM_BP_DIRECT	GO: 0001523~retinoid metabolic process	2	0.025158	LPL, SDC2
GOTERM_CC_DIRECT	GO: 0005886~plasma membrane	5	0.050571	LPL, SLCO4C1, KIT, SLC7A5, SDC2

Table S4 KEGG analysis for intersection genes selected with |logFC|>0.8 for DEM, and |logFC|>1.5 for DEG.

Term	Count	%	P value	Genes
hsa05230: Central carbon metabolism in cancer	2	25	0.036706	KIT, SLC7A5

Table S5 GO analysis for intersection genes selected with |logFC|>1 for DEM, and |logFC|>1 for DEG.

Category	Term	Count	P value	Genes
GOTERM_BP_DIRECT	GO: 2000145~regulation of cell motility	2	0.008311	EGFR, CDK6
GOTERM_BP_DIRECT	GO: 0045429~positive regulation of nitric oxide biosynthetic process	2	0.01274	EGFR, KLF4
GOTERM_BP_DIRECT	GO: 0048146~positive regulation of fibroblast proliferation	2	0.015978	EGFR, CDK6
GOTERM_BP_DIRECT	GO: 0014066~regulation of phosphatidylinositol 3-kinase signaling	2	0.023013	EGFR, IRS1
GOTERM_BP_DIRECT	GO: 0046854~phosphatidylinositol phosphorylation	2	0.027681	EGFR, IRS1
GOTERM_CC_DIRECT	GO: 0005737~cytoplasm	5	0.060267	EGFR, RBMS3, CDK6, IRS1, KLF4
GOTERM_MF_DIRECT	GO: 0046934~phosphatidylinositol-4,5-bisphosphate 3-kinase activity	2	0.018232	EGFR, IRS1
GOTERM_MF_DIRECT	GO: 0005088~Ras guanyl-nucleotide exchange factor activity	2	0.033605	EGFR, IRS1

Table S6 KEGG analysis for intersection genes selected with |logFC|>1 for DEM, and |logFC|>1 for DEG.

Term	Count	%	P value	Genes
hsa05206: MicroRNAs in cancer	3	0.259291	0.005026	EGFR, CDK6, IRS1
hsa04151: PI3K-Akt signaling pathway	3	0.259291	0.007275	EGFR, CDK6, IRS1
hsa05223: Non-small cell lung cancer	2	0.172861	0.024227	EGFR, CDK6
hsa05214: Glioma	2	0.172861	0.028084	EGFR, CDK6
hsa05212: Pancreatic cancer	2	0.172861	0.028084	EGFR, CDK6
hsa05218: Melanoma	2	0.172861	0.03065	EGFR, CDK6
hsa04068: FoxO signaling pathway	2	0.172861	0.057316	EGFR, IRS1
